# Progressive ataxia due to alpha-tocopherol deficiency in Pakistan

**Published:** 2016-04-03

**Authors:** Salman Mansoor, Arsalan Ahmad

**Affiliations:** 1 FCPS Trainee Neurology, Department of Neurology, Shifa International Hospital, Islamabad, Pakistan; 2 Consultant Neurologist, Department of Neurology, Shifa International Hospital, Islamabad, Pakistan

**Keywords:** Vitamin E Deficiency, Ataxia, Alpha-Tocopherol

Ataxia is a common neurological symptom defined as the loss of ability to control and coordinate bodily movements. It has multiple etiologies, among which vitamin E deficiency is a treatable and relatively reversible cause. Early diagnosis of vitamin E deficiency and vitamin E replacement can alleviate the symptoms, and halt the disease progression and even reverse ataxia associated with it. Ataxia resulting from vitamin E deficiency closely resembles Friedreich's ataxia. Patients should be screened for vitamin E deficiency, if they do not possess the diagnostic criteria for Friedreich's ataxia. There have been reports of vitamin E deficiency in Western literature, but a PubMed search did not show any case reports from Pakistan.

This case report presents a 15 year old boy referred to the neurology outpatient department for evaluation of multiple complaints. His complaints started 4 years back with clumsiness in walking which was followed by mood disturbance. After 6 months, his family noted he was being more aggressive which over the course of 4 years slowly progressed. Behavior changes included disinhibition with increased interest in sexuality. He started surfing explicit websites on the internet with pornographic material. He became more intuitive and more curious with increased interest in strangers and their personal belongings. He also had an increased appetite, and in the last 4 years, he had been having progressive weight gain. Past history was significant for paroxysmal nocturnal. 

General examination showed that he had a body mass index (BMI) of 33 kg/m^2^, and his general physical examination was unremarkable. He was fully conscious, alert, and oriented with preserved memory. Gait was ataxic with impaired tandem walking. He had bilateral finger nose ataxia with past pointing. Cranial nerves were intact and vision was normal, pupils were 3 mm round and reactive to light. Ocular movements revealed impaired saccadic eye movements in all directions. Facial sensations and symmetry were normal as were hearing, and tongue and pharyngeal movements. There was no muscle wasting or fasciculations. His muscle tone was normal and power was 5/5 in proximal and distal muscle groups of arms and legs. His ankle reflexes were diminished (1+) with bilateral flexor plantar response. Sensations and joint position sense were normal.

Magnetic resonance imaging (MRI) of the brain showed prominent cerebellar folia (Figure 1). Electroencephalogram (EEG) was essentially unremarkable. Laboratory investigations revealed serum ceruloplasmin of 32 mg/dl (normal: 20-60 mg/dl), serum copper of 142.31 ng/dl (normal: 70-140 ng/dl), serum vitamin E of 2.5 mg/dl (normal: 8.9-18.3mg/l), serum fasting cholesterol of 181 mg/dl (normal: < 200 mg/dl), and serum alpha fetoprotein of 1.84 ng/ml (normal: 0.89-8.78 ng/ml).

**Figure 1:**
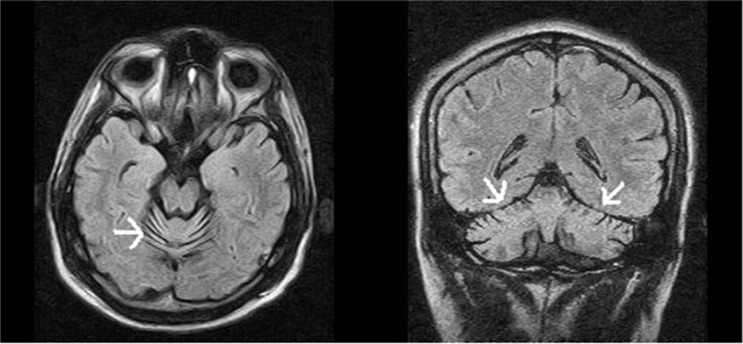
Magnetic resonance imaging (MRI) axial fluid-attenuated inversion recovery (FLAIR) (left) and frontal (right) FLAIR sequences showing prominent folia due to cerebellar atrophy

Serum vitamin E levels showed severe deficiency. Serum electrolytes, serum ceruloplasmin, serum alpha fetoprotein, and vitamin B12 were within normal limits. Moreover, lipid profile and thyroid profile were normal.

He was diagnosed as a case of alpha-tocopherol deficiency. He was prescribed 600 mg vitamin E capsules twice daily. On his subsequent follow-up 3 months later, his behavior had markedly improved with reduced aggressiveness, hypersexuality, and hyperphagia. Yet, his ataxic features still persist. He is currently on vitamin E supplementation (400 mg thrice daily).

Ataxia is a movement disorder characterized by incoordination of movements. Brain structures such as the cerebellum, brainstem, dorsal columns, and vestibular system may be involved. It can be hereditary, sporadic, or secondary to other diseases.^1^

Although Friedreich's ataxia is more common, it can easily be confused with other autosomal recessive cerebellar ataxias (ARCAs) which have similar clinical manifestations. One of the more treatable conditions among these is ataxia with vitamin E deficiency (AVED).

Alpha-tocopherol deficiency is associated with peripheral nerve damage. The condition associated with this deficiency is called AVED. The mechanism underlying this condition is not fully understood. One hypothesis is that vitamin E prevents tissue damage due to oxidative stress.^2^

A hereditary cause of vitamin E deficiency is isolated vitamin E deficiency which manifests as a progressive sensory and cerebellar ataxia that usually begins before 20 years of age. Its clinical signs are similar to those of Friedreich's ataxia which include dysarthria, lower extremity areflexia, loss of proprioception, gait ataxia, and positive bilateral Babinski sign. However, cardiomyopathy and glucose intolerance are usually absent from the clinical presentations of AVED as compared to Friedreich's ataxia.^3^ Thus, in the absence of a frataxin gene mutation, these signs should prompt the physician to consider vitamin E deficiency. As most ataxias are untreatable and progressive, it is necessary to search for causes which are treatable. A timely diagnosis and vitamin E replacement may reverse the condition.^4^ High doses of vitamin E supplementation (800 mg daily) have been shown to reverse the neurological signs of AVED.^4^ In this report, we have highlighted the importance of considering vitamin E deficiency as a cause of progressive but treatable ataxia in children and young adults.

## Notes:

**How to cite this article:** Mansoor S, Ahmad A. Progressive ataxia due to alpha-tocopherol deficiency in Pakistan. Iran J Neurol 2016; 15(2): 103-5.
